# The Frequency of Neuropsychiatric Sequelae After Traumatic Brain Injury in the Global South: A systematic review and meta-analysis

**DOI:** 10.18295/squmj.9.2023.056

**Published:** 2024-05-27

**Authors:** Aishwarya Ganesh, Siham Al-Shamli, Sangeetha Mahadevan, Moon Fai Chan, David T. Burke, Khalid Al Rasadi, Muna Al Saadoon, Samir Al–Adawi

**Affiliations:** 1 Department of Behavioral Medicine, College of Medicine & Health Sciences, Sultan Qaboos University, Muscat, Oman; 3 Department of Family Medicine & Public Health, College of Medicine & Health Sciences, Sultan Qaboos University, Muscat, Oman; 6 Department of Child Health, College of Medicine & Health Sciences, Sultan Qaboos University, Muscat, Oman; 2 Department of Psychiatry, Ibri Hospital, Ibri, Oman; 4 Department of Rehabilitation Medicine, Emory University School of Medicine, Atlanta, Georgia, USA; 5 Medical Research Center, College of Medicine & Health Sciences, Sultan Qaboos University, Muscat, Oman

**Keywords:** Traumatic Brain Injury, Neuropsychiatry, Systematic Review, Meta-analysis, Cognitive Impairment, Anxiety, Depression

## Abstract

This study aimed to assess the prevalence of neuropsychiatric sequelae following traumatic brain injury (TBI) among the Western Asian, South Asian and African regions of the global south. All studies on psychiatric disturbances or cognitive impairment following TBI conducted (until August 2021) in the 83 countries that constitute the aforementioned regions were reviewed; 6 databases were selected for the literature search. After evaluating the articles using the Joanna Briggs Institute guidelines, the random effects model was used to estimate the prevalence of depression, anxiety, post-traumatic stress disorder (PTSD), TBI-related sleep disturbance (TBI-SD), obsessive-compulsive disorder (OCD) and cognitive impairment. Of 56 non-duplicated studies identified in the initial search, 27 were eligible for systematic review and 23 for meta-analysis. The pooled prevalence of depression in 1,882 samples was 35.35%, that of anxiety in 1,211 samples was 28.64%, that of PTSD in 426 samples was 19.94%, that of OCD in 313 samples was 19.48%, that of TBI-SD in 562 samples was 26.67% and that of cognitive impairment in 941 samples was 49.10%. To date, this is the first critical review to examine the spectrum of post-TBI neuropsychiatric sequelae in the specified regions. Although existing studies lack homogeneous data due to variability in the diagnostic tools and outcome measures utilised, the reported prevalence rates are significant and comparable to statistics from the global north.

A widely accepted definition of traumatic brain injury (tbi) is yet to be established.^1^ However, concisely, TBI is a condition that results from external mechanical forces injuring brain tissues and compromising the integrity of brain functioning. The outcome is a cascade of biopsychosocial disturbances that lead to transient or permanent functional outcomes.^1^–^4^ Among the various neuropsychiatric sequelae of TBI are cognitive, emotional, behavioural and sensorimotor disturbances. The frequency of behavioural and emotional disturbances has been extensively studied, with Ponsford *et al*. reporting that 18.3–83.3% of those who sustain TBI have these outcomes.^5^ This wide variation in the rate of post-traumatic secondary conditions is likely due to many factors, including the time since the injury, the diagnostic tool used and the quantification of the severity of the TBI and case ascertainment.^6^^,^^7^

According to the Diagnostic and Statistical Manual of Mental Disorders (DSM), mild and severe neurocognitive disorders due to TBI both have the potential to contribute to dependency and disability.^8^–^10^ TBI coupled with secondary neuropsychiatric symptoms tends to account for the greater part of the cost of healthcare utilisation.^11^–^13^ Studies have reported that a critical predictor of poor psychosocial outcomes following TBI is the initial level of cognitive or functional impairment.^14^^,^^15^

Globally, approximately 69 million people sustain a TBI each year.^16^ Lower-middle-income countries in the global south have shown a prevalence of TBI of 811/100,000 population.^16^ However, this rate could just be the superficial indication of a broader, underlying issue since there is a lack of high-quality data in these regions.^16^^,^^17^ The mortality and disability rates after TBI in these countries is high, representing one-third to half of all trauma-related deaths and injuries in the world.^18^ The majority of those injured are in their prime productive years between the ages of 11 and 40.^18^^,^^19^

Although it is inappropriate to paint all developing countries with the same broad strokes, the healthcare issues common to several of these countries include infectious and environmental diseases, high infant mortality rates and lack of food security. However, non-communicable diseases and associated long-standing health concerns are gaining importance, with recent estimates suggesting that 2.4 billion people have a disability, and 49 million of these have a disability attributable to TBI.^20^^,^^21^ Despite the increasing tide of non-communicable diseases, such as TBI, in Western Asian, South Asian and African countries, efforts have generally been geared toward cure-oriented biomedical care for communicable diseases. TBI is often relegated to the sphere of minor health concerns by government healthcare planners, giving it the characteristic trait of a ‘silent epidemic’.^16^

Data suggest that TBI-related mortality during hospitalisation is decreasing, particularly in the global north.^22^ Improved outcome rates can be largely attributed to access to specialised intensive care units, which is often unavailable to those of lower socioeconomic status living in developing countries with scarce resources.^23^ While TBI affects all age groups, detailed analyses have shown that its occurrence follows a trimodal distribution, often occurring more in children, young adults and senior citizens.^24^^,^^25^ Many countries in the global south are thought to be in the second phase of demographic transition, where there is a high birth rate and an increasing life span.^26^ These demographic changes have heightened the concentration of the ‘youth bulge’ in the population structure, which also correlates with an increased use of automobiles.^27^^,^^28^ Due to this increased exposure to risk factors and sparse healthcare resources, countries in the global south are likely to experience a higher burden of TBI compared to those in the global north.^16^

These findings are especially necessary to consider due to some of the significant differences in the prevalence of TBI between the global south and north. One key distinction is the epidemiology of TBI; Africa and Southeast Asia report the highest incidence rates of TBI to be among younger demographics due to ‘road traffic accidents’, while North America reports falls in the elderly as a significant cause.^18^ People from the global south also have twice the odds of death after a severe TBI compared to their counterparts in the global north.^29^ The majority (93%) of the TBI prognostication models in use are also based on samples from the global north.^30^ These are significant factors that call for management protocols that are sensitive and specific to these demographically distinctive groups.

With these factors in mind, it is important to note the lack of systematic reviews and statistics on TBI and its related adverse short- and long-term neuropsychiatric outcomes in Western Asia, South Asia and Africa, which are all regions of the ‘global south’.^16^ A study by Tropeano *et al*. reflects this trend, indicating that a higher proportion of studies evaluating the burden of TBI are conducted in countries in the global north, despite the fact that approximately 80% of the world population resides in the global south.^31^^,^^32^

This systematic review and meta-analysis aimed to assess the prevalence of psychiatric symptoms and cognitive impairment following TBI, specifically in the Western Asian, South Asian and African regions of the global south. It is essential to consider psychiatric symptoms and cognitive impairment in tandem because of the bidirectional relationship between them with respect to aetiology, presentation and treatment. A critical evaluation of existing literature on the magnitude of neuropsychiatric disturbances in the post-TBI population will help to lay the groundwork for evidence-based management and rehabilitation promotion programmes such as the World Health Organization’s (WHO) Rehabilitation 2030.^33^ The ‘global south’ is a geopolitical term used as a shorthand to denote economically, politically or culturally marginalised regions outside of Europe and North America.^34^ Although the global south is a vast region that includes South and Latin America, the Pacific Islands, Africa and Asia, for brevity, the present review of the prevalence of neuropsychiatric complications after TBI will focus specifically on western and southern Asia and Africa.

## Methods

The present systematic review was conducted in accordance with the PRISMA (Preferred Reporting Items for Systematic Reviews and Meta-Analyses) guidelines and included all articles published and in print up to August 2021.^35^ The article-extraction process began with the use of search terms across different levels, delimited using the Boolean operators ‘AND’ and ‘OR’. The first level (for TBI) included search terms such as ‘Traumatic brain injury’ OR ‘head impact’ OR ‘brain injury’. Level 2 (for psychiatric and cognitive symptoms) included the following search terms: ‘mental disorder’ OR ‘psychiatric disorder’ OR ‘mental illness’ OR ‘cognitive impairment’ OR (other specific individual mental disorders such as ‘depression’, ‘anxiety’, ‘eating disorders’, ‘PTSD’, ‘dementia’, ‘cognitive decline’, etc.). The final level included the individual country names (Gulf Cooperation Council: Oman, Kuwait, Bahrain, Saudi Arabia, Qatar and the United Arab Emirates; Western Asia: Israel, Iraq, Jordan, Palestine, Lebanon, Iran, Syria, Afghanistan, Pakistan, Bahrain, Kuwait, Qatar, Oman, United Arab Emirates, Saudi Arabia and Yemen; South Asia: Bhutan, Bangladesh, Pakistan, India, Sri Lanka, Nepal, Afghanistan and the Maldives; Africa: Algeria, Angola, Botswana, Benin, Burundi, Burkina Faso, Cabo Verde, Central African Republic [CAR], Cameroon, Comoros, Chad, Republic of Congo, Democratic Republic of Congo, Djibouti, Cote d'Ivoire, Egypt, Equatorial Guinea, Eswatini [formerly Swaziland], Eritrea, Gabon, Ethiopia, Ghana, Gambia, Guinea–Bissau, Guinea, Lesotho, Kenya, Libya, Liberia, Malawi, Madagascar, Mali, Mauritius, Mauritania, Mozambique, Morocco, Niger, Namibia, Nigeria, Rwanda, Sao Tome and Principe, Seychelles, Senegal, Somalia, South Africa, Sierra Leone, South Sudan, Sudan, Togo, Tanzania, Tunisia, Zambia, Uganda and Zimbabwe). The accumulated articles were further screened to ensure that they met the eligibility criteria. This systematic review was registered with PROSPERO (registration ID CRD42021270604).

### DATA-RETRIEVAL STRATEGIES

Based on the inclusion criteria, the process of article identification began with a complete screening of major databases—PsycINFO, Scopus, PubMed/MEDLINE, ProQuest for English articles and the Al-Manhal database for Arabic articles—by three independent researchers (AG, SS and SM). A final search of up to 10 pages on Google Scholar was also performed to ensure the inclusion of any articles (including grey literature) that may have been missed. This aforementioned search strategy did not include a search based on a specific timestamp, implying that any and all articles, including those published or in press as at August 2021, were included in the search.

The full versions of the articles were downloaded if their titles and abstracts met the inclusion criteria. After excluding the articles that did not meet the inclusion criteria, the three independent researchers (AG, SS and SM) produced a total of 52 articles for quality review using the Joanna Briggs Institute (JBI) guidelines—the prevalence checklist—for the evaluation of scientific research articles.^36^ In case of a disagreement between the three main researchers, the other 3 researchers (SA, MS and MFC) were consulted for a discussion until a consensus was reached.

### INCLUSION AND EXCLUSION CRITERIA

The inclusion criteria were as follows: (1) original research (newly conducted studies or studies that used secondary data); (2) studies which included civilian populations in their samples; (3) studies that measured some form of psychiatric disorder or cognitive impairment after a single TBI, using standardised diagnostic procedures or self-reported measures, regardless of the time interval following the TBI event; (4) prospective or retrospective cross-sectional, cohort or case–control studies; (5) studies written in English or Arabic; and (6) studies from Western Asia, South Asia and Africa.

Studies were excluded if: (1) their samples included military personnel and war veterans; (2) the participants reported a TBI that had not been diagnosed in a medical setting (i.e. diagnosed based on non-standardised measures and methods); (3) the participants had a psychiatric illness, cognitive impairment, intellectual disability, or other neurological events prior to the TBI; (4) they are reviews, case studies, case reports, brief reports, brief communications or any other type of article besides an original research; and (5) they only reported average scores for psychometric measures but not prevalence.

The current study’s population comprised civilians who had been appropriately diagnosed with a TBI, as gleaned through the guidelines of the Federal Interagency Traumatic Brain Injury Research Informatics System for TBI Research (2015), the American Congress of Rehabilitation Medicine (1993), the Department of Veterans Affairs and the Department of Defence (2009) and the International and Interagency Initiative toward CDE for Research on TBI and Psychological Health (2010).^1^–^4^ Although there was no homogeneous agreement on the exact evaluative procedures used for the diagnosis of TBI, the condition generally involved damage to or infarction of brain tissues attributable to an external mechanical force, as evidenced by loss of consciousness, posttraumatic cognitive and behavioural changes or any other objective neurological finding.^37^

### EVALUATION OF THE QUALITY OF STUDIES

According to the standardised items listed in the JBI checklist for prevalence studies, the three reviewers independently evaluated the title, abstract, methods, results, discussion and other sections of each included study.^38^ The resulting interrater reliability of the 3 independent reviewers of the current quality measure was strong, with an intraclass correlation coefficient of 0.88. After completely evaluating the articles using the JBI checklist, the reviewers had to decide which articles were of sufficient quality to be included in the systematic review and data extraction process. No single approach is considered best practise. Porritt *et al*. recommended mutual agreement between the members of the research team.^39^ Since the JBI checklist consists of 9 questions, each article was scored on a scale of 0–9. It was decided among the researchers that the articles that earned a score of ≥7 would be included in the systematic review and data extraction process.

### DATA EXTRACTION

Three independent researchers (AG, SS and SM) extracted relevant information from the included studies, such as the name of the first author; the year of publication; the duration of the study; the country in which the study was conducted; the sampling methods used; the median, mean and standard deviation of the age of participants along with their age range; the characteristic of the sample (university student, patient, etc.); the sample size; the gender distribution of the sample; the assessment tools used; the reliability of the said tools; the disorder screened; the total number of positive cases and the duration after which neuropsychological tests were administered (post-TBI duration).

### PATIENT AND PUBLIC INVOLVEMENT

There was no direct patient or public involvement or recruitment for the purpose of this study.

### STATISTICAL ANALYSIS

The acquired data were analysed using the MedCalc 12 statistical software (MedCalc Software Ltd, Ostend, Belgium). In this review, 6 main psychological outcomes of patients with TBI were identified: depression, anxiety, post-traumatic stress disorders (PTSD), obsessive–compulsive disorders (OCD), TBI-related sleep disturbance (TBI-SD) and cognitive impairment. In the meta-analysis, the estimated pooled prevalence for each outcome was calculated.^40^ The I^2^ and Q statistics were used to assess heterogeneity between articles with the same outcome.^41^ The 95% confidence interval (CI) of each study was estimated using the binomial method available in the MedCalc software (MedCalc Software Ltd). For the heterogeneity test, a random effects model was used to interpret the results if the I^2^ statistic was >50% and the Q statistic was <0.1; otherwise, the researchers used the fixed effects model.^41^^,^^42^

## Results

An initial search of the databases yielded a total of 166 usable articles. Subsequently, duplicates (n = 9) and inaccessible (n = 3) articles as well as articles that did not meet the inclusion criteria (n = 104) were removed, leaving a total of 50 articles. Another 6 articles were added after searching the references of the included articles giving a total of 56 articles included in the analysis [Supplementary Figure 1].

Of the 56 unduplicated original studies identified by the initial search, 27 (which earned a score ≥7, according to the JBI criteria) were considered eligible for the systematic review [Supplementary Table 1].^37^^,^^43^–^68^ Four studies were further excluded because they could not be grouped into any category based on symptoms, as each study covered a unique disorder (i.e., post-concussive syndrome or symptoms, aggression and post-traumatic amnesia). Finally, 23 studies were used for the meta-analysis [Supplementary Figure 1].^37^^,^^43^–^64^

Although the initial database search included 83 countries, only 27 studies from the following 10 countries were finally included in the study: Israel, Iran, Oman, Morocco, India, Nepal, Tunisia, Ethiopia, Nigeria and Uganda [Supplementary Figure 1 and Table 1].

The highest number of studies came from India (n = 12), followed by Iran (n = 5) and then Oman and Israel (with 2 studies each); the remaining countries—Morocco, Nepal, Tunisia, Ethiopia, Nigeria and Uganda—only produced 1 study each. The neuropsychological symptoms reported in the studies included depression (16 studies), anxiety (11 studies), PTSD (3 studies), OCD (3 studies), TBI-SD (4 studies) and cognitive impairment (8 studies).

**Figure 1:**
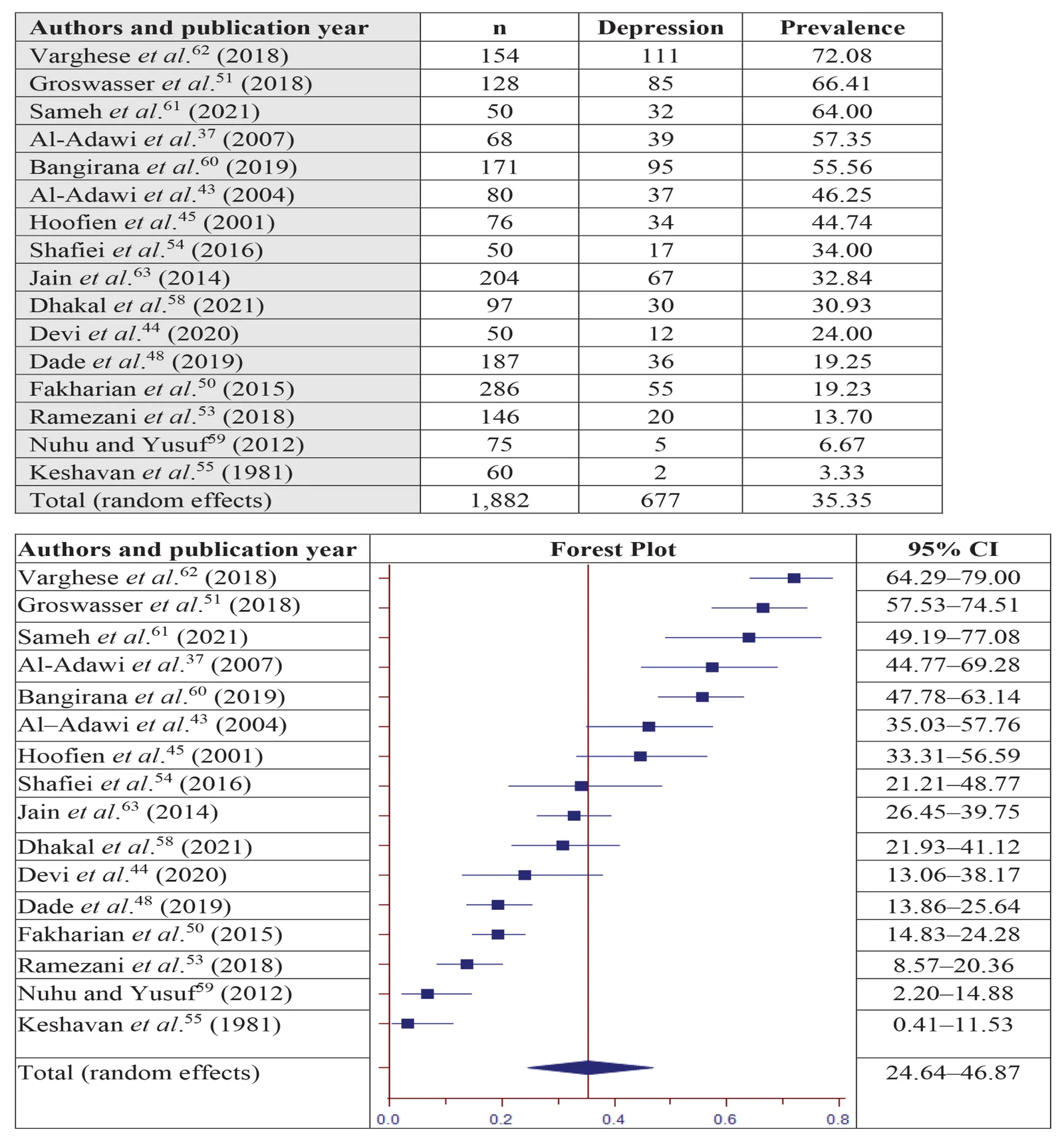
Prevalence estimates of depression following traumatic brain injury (N= 1,882). *Heterogeneity: I^2^ = 96.20%, Q = 394.96; P <0.001*. CI = confidence interval

The pooled prevalence of depression in a total sample of 1,882 was 35.35% (95% CI: 24.64–46.87%), based on the random effects model (I^2^ = 96.20%, Q = 394.96; *P* <0.001) [Figure 1].

**Figure 2:**
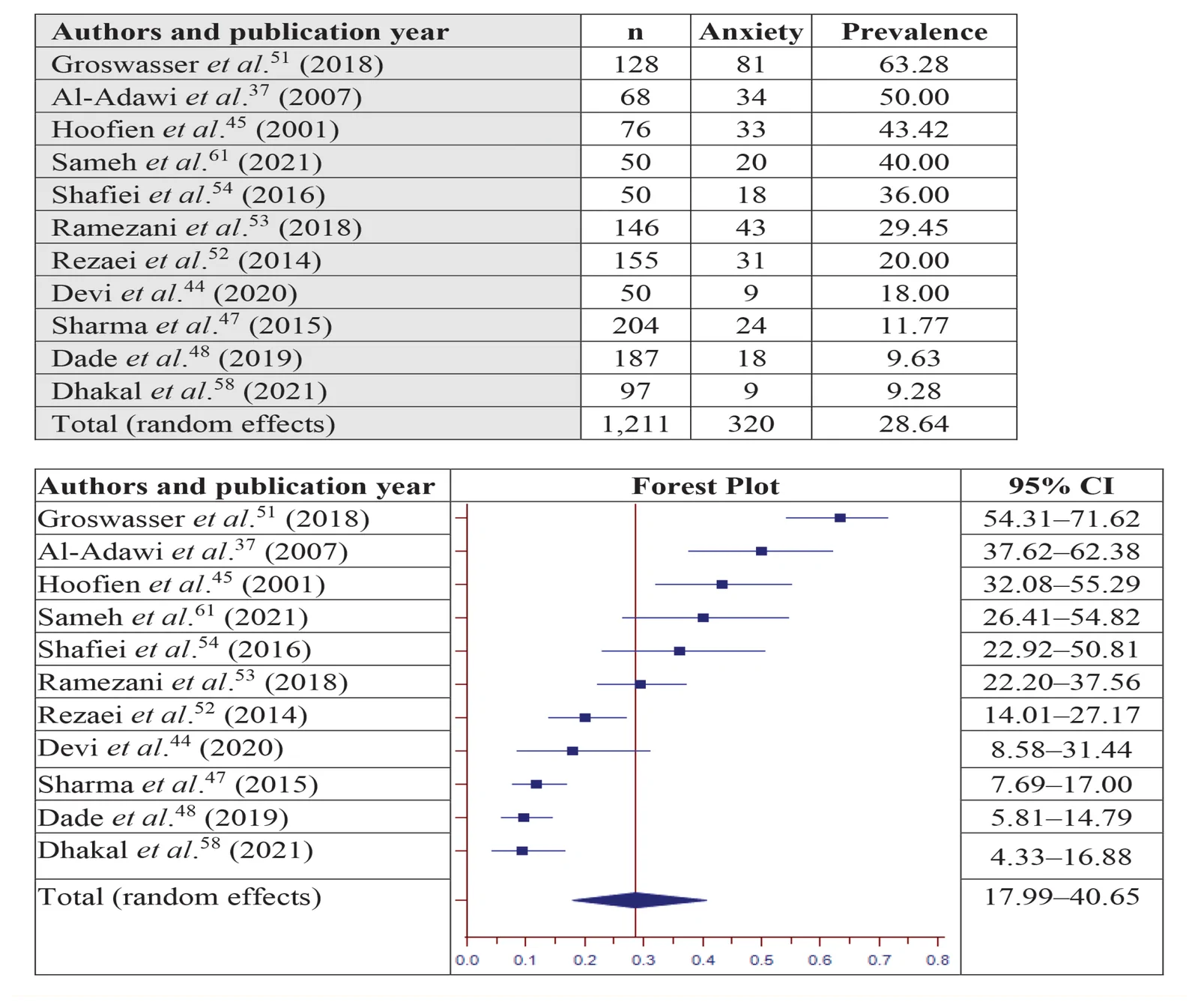
Prevalence estimates of anxiety following traumatic brain injury (N = 1,211). *Heterogeneity: I^2^ = 94.92%, Q = 196.91; P <0.001*. *CI = confidence interval*.

The pooled prevalence of anxiety in a total sample of 1,211 was 28.64% (95% CI: 17.99–40.65%), based on the random effects model (I^2^ = 94.92%, Q = 196.91; *P* <0.001) [Figure 2].

**Figure 3:**
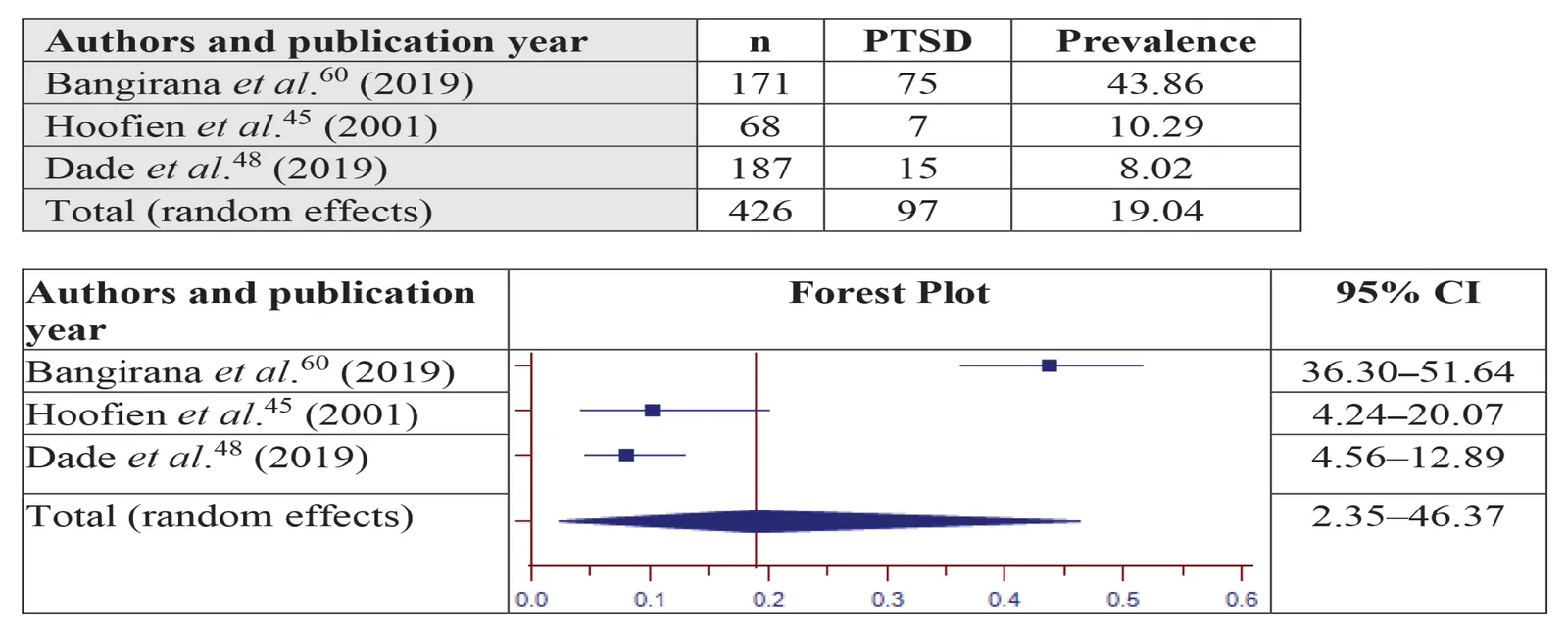
Prevalence estimates of post-traumatic stress disorder following traumatic brain injury (N = 426). *Heterogeneity: I^2^ = 97.28%, Q = 73.46; P <0.001*. *CI = confidence interval; PTSD = post-traumatic stress disorder*.

The pooled prevalence of PTSD in a total sample of 426 was 19.04% (95% CI: 2.35–46.37%), based on the random effects model (I^2^ = 97.28%, Q = 73.46; *P* <0.001) [Figure 3].

**Figure 4:**
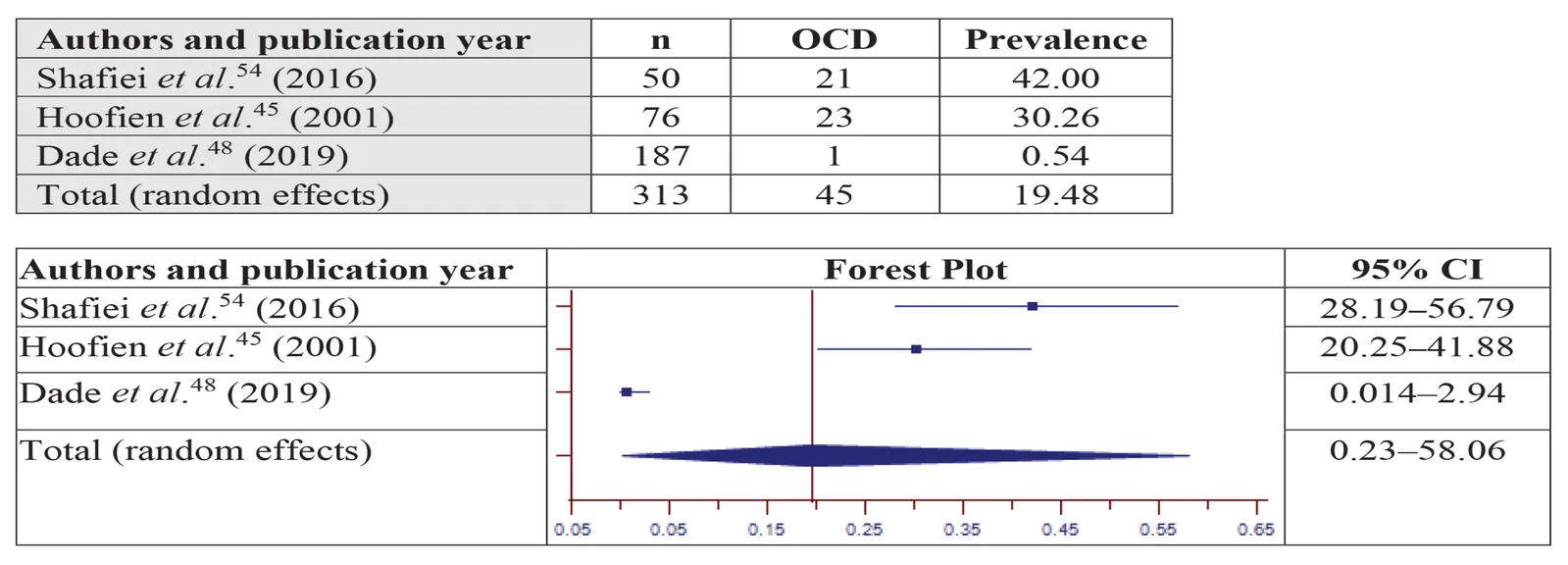
Prevalence estimates of obsessive-compulsive disorders following traumatic brain injury (N = 313). *Heterogeneity: I^2^ = 97.84%, Q = 92.44; P <0.001*. *CI = confidence interval; OCD = obsessive-compulsive disorder*.

The pooled prevalence of OCD in a total sample of 313 was 19.48% (95% CI: 0.23–58.06%), based on the random effects model (I^2^ = 97.84%, Q = 92.44; *P* <0.001) [Figure 4].

**Figure 5:**
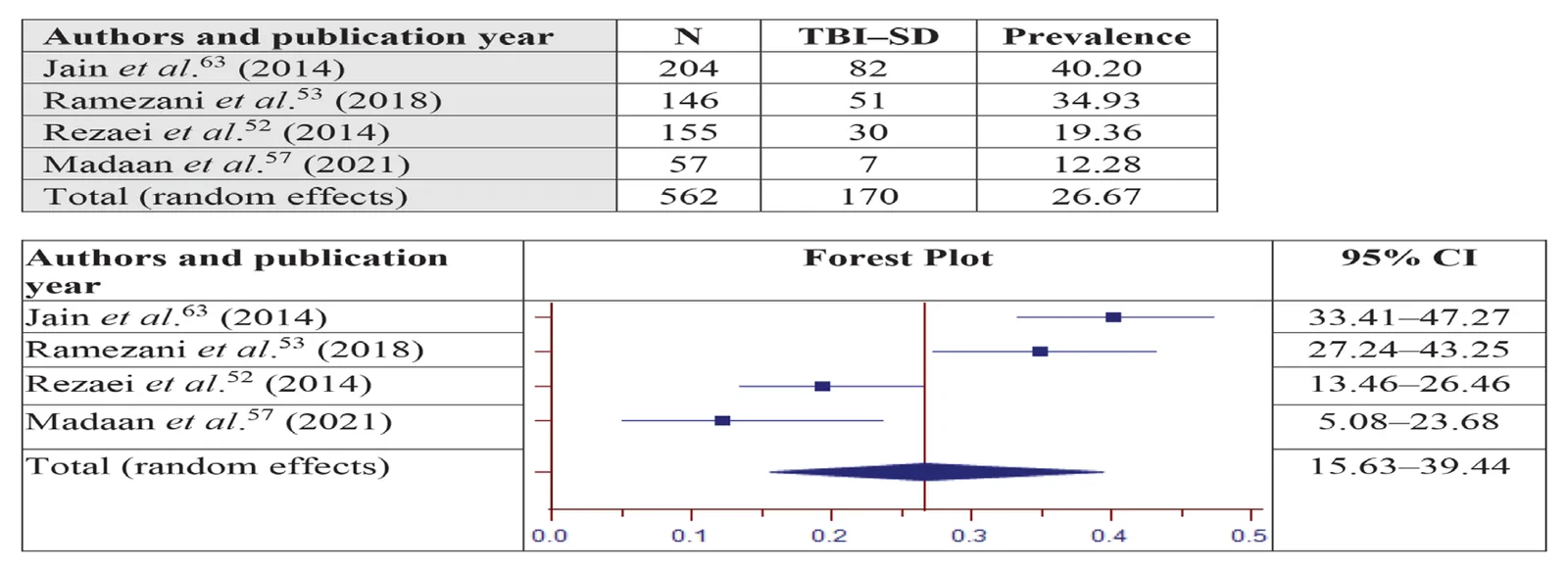
Prevalence estimates of traumatic brain injury-related sleep disturbance (N = 562). *Heterogeneity: I^2^ = 90.27%, Q = 30.83; P <0.001*. *CI = confidence interval; TBI-SD = traumatic brain injury-related sleep disturbance*.

The pooled prevalence of SD in a total sample of 562 was 26.67% (95% CI: 15.63–39.44%), based on the random effects model (I^2^ = 90.27%, Q = 30.83; *P* <0.001) [Figure 5].

**Figure 6:**
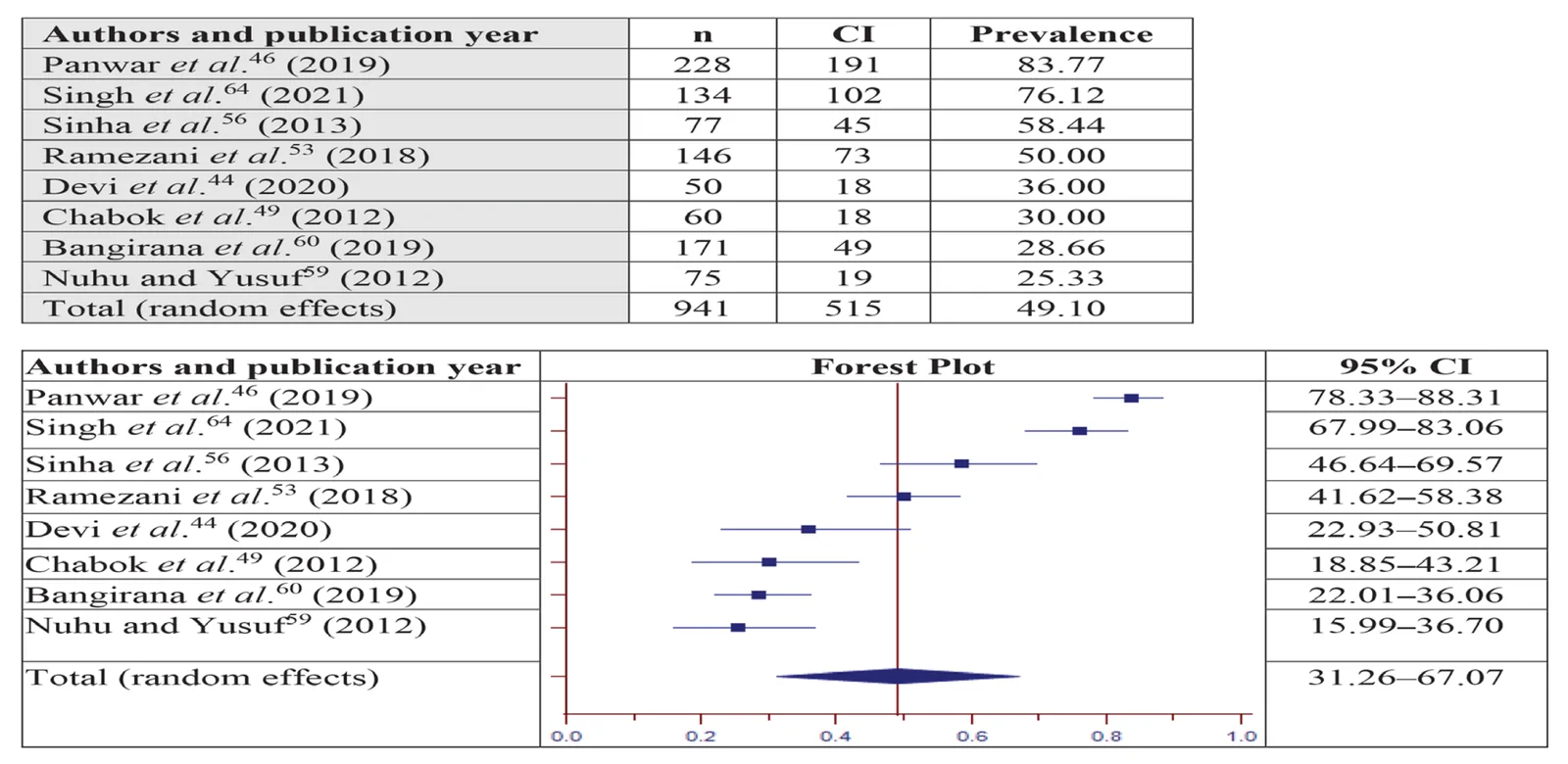
Prevalence estimates of cognitive impairment following traumatic brain injury (N = 941). Heterogeneity: I^2^ = 96.85%, Q = 222.41; *P* <0.001. CI = confidence interval.

The pooled prevalence of cognitive impairment in a total sample of 941 was 49.10% (95% CI: 31.26–67.07%), based on the random–effects model (I^2^ = 96.85%, Q = 222.41; *P* <0.001) [Figure 6].

## Discussion

To lay the groundwork for the possible evolution of healthcare systems in the global south to address ‘silent epidemics’ such as TBI, in addition to programmes such as the WHO's Rehabilitation 2030, the current systematic review and meta-analysis aimed to critically evaluate the prevalence of the cognitive and psychiatric sequelae of TBI, specifically in Western Asia, South Asia and Africa.^20^^,^^33^ High TBI prevalence leads to significant mortality and disability rates, amplified by healthcare challenges and limited resources. Despite the rising trend in non-communicable diseases, healthcare priorities still favour communicable diseases. TBI outcome improvements in the global north due to specialised care contrast with the resource limitations of the global south. Demographic shifts and a distinct TBI epidemiology contribute to a higher burden in this region. TBI burden persists with inadequate research and statistics in the global south, necessitating tailored management approaches.

The current analysis suggests that the prevalence of depressive symptoms derived from 16 studies is 35.35%. Among the studies used to assess the prevalence of depression, a distinction needs to be made between those that used self-reporting measures and those that used standardised diagnostic procedures to deduce the presence of depressive symptoms and disorders. Most of the studies employed tools such as self-report measures, which tap into subthreshold depressive or negative symptoms, providing spurious results [Supplementary Table 1].

Osborn *et al*. compared the influence of the type of diagnostic measure used on the prevalence rates of depression in an Australian sample.^69^ In their study, 27% of the sample were formally diagnosed using standardised procedures, while 38% reported clinically significant depressive symptoms using self-report measures.^69^ The prevalence rate of depression in the current study—35.35%—falls between the two figures from the study by Osborn *et al*. Furthermore, in a systematic review, Scholten *et al*. reported that the pooled prevalence estimate of depressive disorders was 17% in the first year after TBI and that a higher long-term prevalence of 43% was observed.^70^ These results suggest that the expression of depressive symptoms fluctuates in a complex way, depending on whether the symptoms were diagnosed using self-report measures or standardised diagnostic procedures and on the time interval between the TBI event and diagnosis. More studies are needed to clearly demarcate between depression and negative symptoms such as psychomotor retardation, fatigue, apathy, anhedonia and abulia.^71^

In the current review, the estimated prevalence of anxiety-related disorders following TBI in 11 studies stood at 28.64%. Anxiety disorders were mostly diagnosed using self-report measures; therefore, it is important to consider the possible inflation of the reported prevalence rate [Supplementary Table 1].

A meta-analysis by Osborn *et al*. compared the outcome measures used and the time interval since the TBI and reported that 11% of the sample were diagnosed with general anxiety disorders (GAD) when using standardised diagnostic procedures, while 37% were diagnosed with GAD when using self-report measures.^72^ Scholten *et al*. conducted a systematic review of the prevalence of anxiety symptoms and reported that the pooled prevalence estimate of anxiety was 21% in the first year following TBI.^70^ Therefore, it is likely that factors that impact depressive symptoms also play a role in the expression of anxiety symptoms. Furthermore, Gould *et al*. reported that a pre-injury diagnosis of anxiety-related disorders increased the probability of having a post-TBI anxiety disorder, the prevalence of which progressively increased each month after trauma.^73^ Therefore, demographic variability in the general prevalence of anxiety-related disorders is likely to also impact the post-TBI diagnosis of GAD.^74^^,^^75^ Concerted efforts are needed to establish a robust data collection method that accounts for such confounders in this region.

Statistics related to PTSD in populations of interest are often considered controversial due to inaccurate reporting or interpretation of responses using self-reporting questionnaires, as well as the questionable cross-cultural applicability of the concept of PTSD featured in the DSM and International Classification of Diseases (ICD).^76^^,^^77^

In the current study, the estimated prevalence of PTSD derived from three articles was 19.04%. A systematic review and meta-analysis by Van Praag *et al*. reported the prevalence of PTSD after TBI to range between 0% and 36%, with a pooled prevalence rate of 15.6%.^78^ Another systematic review and meta-analysis by Iljazi *et al*. captured the longitudinal fluctuation of PTSD symptoms after TBI, reporting that the prevalence rate was 2.2% after 3 months, 16.3% after 6 months, 18.6% after 12 months and 11.0% after 24 months.^79^ Such an analysis is better equipped to distinguish between an acute adjustment disorder and full-fledged PTSD. The current study’s reported prevalence of 19.04% falls within the prevalence range reported in the studies mentioned above. However, not all the selected studies accrued in the current systematic review revealed the ‘time since TBI’ and the presentation of symptoms of PTSD, making it impossible to assess the longitudinal relationship between these 2 factors.

The pooled prevalence of OCD from the 3 relevant studies in the current review stood at 19.48%. In the general population, OCD reportedly has a prevalence rate of about 2.3%, a number that supposedly transcends ethnicity and geography.^80^^,^^81^ Unlike many psychiatric disorders that are likely to be stigmatised in many traditionally religious societies that subscribe to scriptural teachings, a high level of health seeking behaviour, in both biomedical and traditional healing settings, has been observed for OCD.^82^^,^^83^ It has been hypothesised that the focus on purity, cleanliness, thought control, morality and sexuality could trigger the development of OCD.^84^ In the general population, OCD has been associated with abnormalities in the frontostriatal region of the brain, an anatomical region that often undergoes microstructural damage due to TBI.^85^^,^^86^ Rydon-Grange and Coetzer suggested that OCD secondary to TBI tends to be ‘masked’ as cognitive impairment and that, conversely, memory impairment and executive dysfunction are often incorrectly diagnosed as OCD.^87^^,^^88^ Given this context, more studies are needed to discern whether OCD and the other sequelae This sentence is incomplete. Please check it.

The current study revealed an estimated pooled TBI-SD prevalence of 26.67% from 4 studies. TBI-SD can hamper the recovery process of TBI, as well as potentially increase the incidence of various comorbidities, including the post-TBI spectrum of neuropsychiatric impairment.^89^–^91^ Reciprocally, mood and anxiety disorders, along with more direct factors such as the degree of injury to regions of the brain involved in sleep—the hypothalamus, brainstem and reticular activating system—can also contribute to the development of sleep disturbances.^92^ Mathias and Alvaro identified hypersomnia, insomnia, narcolepsy, obstructive sleep apnoea and periodic limb movements as the most common sleep problems associated with TBI.^93^ A systematic review and meta-analysis by Montgomery *et al*. reported the pooled prevalence of insomnia disorder to be 27.0%, which closely resembles the prevalence rate found in the current study.^94^ Given that TBI-SDs are considered one of the most prevalent and persistent sequelae of TBI, more studies with larger samples are required to explore the complex interconnections between post-TBI sleep–wake patterns and other neuropsychiatric complications.

In the current study, the estimated prevalence of cognitive impairment from 8 studies was 49.10%. Unlike post-traumatic psychiatric disorders, cognitive impairments that affect memory, sensorimotor and functional status have been widely established to be strongly associated with damage to specific areas of the brain. Impaired cognition is associated with difficulties in information processing, resulting in problems with attention and concentration, learning and remembering, executive functioning and other higher-order functions that fall under the rubric of neuropsychological impairment. A meta-analysis of the prevalence rate of cognitive deficits after TBI reported a pooled prevalence of 18–57%.^95^ This wide variation probably stemmed from the excessive heterogeneity of the time of cognitive assessment (acute versus chronic) and the severity of injury (moderate versus severe). The prevalence rate obtained in the current review falls within the range reported in the abovementioned meta-analysis. The presence of cognitive decline has the potential to negate self-sufficiency, creating subtle but intransigent disability and dependency.^96^

### LIMITATIONS

Kim *et al*., exploring whether published studies on post-TBI neuropsychiatric sequelae met the criteria of the American Academy of Neurology for the classification of articles on diagnostic methods, identified one limitation of their study as the rarity of articles on this subject that employed a robust methodology with usable data.^97^ Similar conclusions were also drawn when the articles included in this study were analysed. Unfortunately, certain high-quality articles had to be excluded from the meta-analysis, as many of them reported prevalence data as continuous measures (i.e. they reported scores as means). Furthermore, as is often the case, systematic reviews and meta-analyses tend to have their own intrinsic conceptual and methodological limitations. These potential limitations will be discussed, along with a critical appraisal of the studies from the regions of interest—West and South Asia and Africa.

### HETEROGENEITY OF OUTCOME MEASURES

For logistical reasons and due to the excessive heterogeneity of the tools used, it was not feasible to distinguish articles based on their outcome measures. Thus, the ideal model of grouping prevalence rates according to whether they used self-report measures or standardised diagnostic procedures was not feasible in the current review. On one hand, to avoid false comparisons, it is often ideal to calculate the prevalence rate using specific outcome measures. However, the method of lumping itself has limitations. As is often the case, self-report measures and standardised diagnostic procedures both tend to reveal significant differences in prevalence rates, with standardised diagnostic procedures tilting towards more conservative figures. The current review has the confounder of not being able to separate apples from oranges; therefore, caution is needed when interpreting its findings. Similarly, it would have been ideal if the studies in the regions considered in the current review had quantified psychiatric symptoms which are part of the international psychiatric nosology. For example, some studies used the Self-Reported Questionnaire (SRQ), and while this questionnaire has been specifically designed by the WHO for non-western populations, it only detects non-specific psychological distress. However, Bangirana *et al*. used it to tap into depressive symptoms.^60^ In addition to the SRQ, other instruments such as the General Health Questionnaire, Apathy Evaluation Scale and Brief Symptom Inventory appeared to have been used to tap into psychological problems and symptoms of psychopathology that are not commonly used for rigorous neuropsychological evaluation. However, such measures have various subscales that measure distresses featured in the DSM and ICD, such as the study by Devi *et al*. utilizing the Neuropsychiatric Inventory Questionnaire, which is an informant-based instrument.^44^^,^^98^ Therefore, a differentiation in terms of whether these instruments are capable of measuring specific functional outcomes and psychiatric and cognitive symptoms is needed.

### PROBLEMS RELATED TO THE ASSESSMENT OF COGNITION

While cognitive impairment following TBI is common, there is currently no widely accepted, unified process of quantifying it. In the articles reviewed in this study, the tools used to assess cognition are those considered to be ‘bedside’ global cognitive tests, rather than conventional neuropsychological batteries.^99^ They frequently produce false positives, depending on the patient's education status, as well as false negatives, depending on the anatomical region of the brain injury.^100^ Similarly, important confounders of cognitive functioning, such as language proficiency, premorbid intelligence quotient and mood status, were not adequately addressed in studies conducted in the regions of interest.

### THE RELATIONSHIP BETWEEN COGNITIVE AND PSYCHIATRIC SYMPTOMS

Some emotional distress and affective symptoms are likely to have a reciprocal relationship with cognitive symptoms. Similarly, individuals’ premorbid functioning and level of education have been widely established to influence their post-TBI cognitive status. These relationships were not explored in-depth in the articles from the region under study.

### TIME SINCE INJURY

Longitudinal studies show fluctuating prevalence rates of secondary conditions following TBI.^79^^,^^101^ However, most of the articles that met the inclusion criteria for this study did not explicitly mention the time since injury, making it impossible for the authors to categorise and evaluate the results based on the time since injury.

### DIVERSITY IN LANGUAGE

The regions considered are known for their diverse spoken languages, some of which include Hindi, Farsi, Hebrew, Urdu, Arabic and Swahili. Although attempts were made to access the TBI literature in Arabic through the Al-Manhal database (to no avail), the authors of the current review could not evaluate any non-English-language articles that may exist.

### HETEROGENEITY OF THE INCLUSION AND EXCLUSION CRITERIA

Most of the articles included in the current review did not indicate the specifics of the diagnostic criteria for TBI within their inclusion and exclusion criteria. What constitutes TBI is sometimes wrongly equated with perinatal trauma, hypoxia-ischaemia events, cerebral oedema, toxic and metabolic insult, primary ischemic or haemorrhagic strokes, seizure or its aftermath, intracranial surgery, cerebral neoplasms, skull fracture and intracranial haematoma without concurrent cerebral injury.

### REGIONS OF CONFLICT

It must be noted that a few of the countries included in the current review are at present, or have been, settings of major military conflicts. Although studies on TBI among military personnel were excluded, there were no internal mechanisms to rule out combat or war-related incidents in non-military samples. For example, blast-induced TBI is a unique diagnosis that has been identified as a characteristic cause of injury resulting from conflicts in Iraq and Afghanistan due to the different physical attributes and biological consequences that make it significantly different from other modes of injury.^102^

### POTENTIAL DUPLICATION OF DATA

In any given region, many of the studies on this topic have been performed by a similar set of authors using data from the same one or two healthcare settings in the region. Therefore, it was not possible for the authors to account for the potential duplication of data in research articles analysing various psychiatric and cognitive symptoms.

### DATA POLLUTION

Unlike data poisoning, which refers to ‘intentional attempts to feed inaccurate data into models’, data pollution is the unintentional corruption of data due to various reasons, such as poor measurement reliability, amorphous or heterogeneous definitions of key concepts and selection bias.^103^ There is a chance that data pollution affected the current review’s data, given the heterogeneous nature of the data, the lower quality of the sample selection procedures employed in the included studies and the use of self-reported measures.

### PUBLICATION BIAS

It is recommended that a publication bias assessment be done to account for any potential outliers and this was adhered to in the current study by conducting a search for any grey literature on the all-inclusive database Google Scholar. Additionally, preventing publication bias also requires that studies included in a high-quality meta-analysis be better powered. However, most of the papers in the region of interest that met the inclusion criteria of the current meta-analysis did not provide a proper explanation for the calculation of their sample size. This may likely be because adherence to reporting guidelines, such as the ‘Strengthening the Reporting of Observational Studies in Epidemiology’ (STROBE) guidelines, is generally suboptimal in studies in the regions under consideration.^104^ Given the limited availability of existing research on this study’s topic, the authors decided to include articles that provide prevalence rates relevant to the current study. Therefore, this particular aspect of publication bias was overlooked.

### OVER-REPRESENTATION OF CERTAIN REGIONS

Among the countries in the region of interest, 2 (India and Iran) were over-represented, contributing 17 of the 27 included studies. Although Western Asian countries did produce a reasonable amount of research publications, unfortunately, several fell short of the standards of the JBI guidelines. Concerted efforts are needed for TBI research to thrive in these regions, especially since their populations are known to be at higher risk.^33^

### SPECIFICITY OF THE PRESENTING SYMPTOMS

Survivors of TBI frequently exhibit a range of neuropsychiatric symptoms, often described using the term ‘postconcussion syndrome’. This syndrome is characterised by a confluence of cognitive, emotional, behavioural and even physical issues. This amalgamation of symptoms contributes to the intricate nature of their diagnosis. However, the intricate nature of this diagnostic spectrum introduces complexities to comprehending these conditions. The labels and classifications applied to these conditions are significantly influenced by the specific screening tools used for assessment. Consequently, these variations in labelling can substantially affect the estimated prevalence rates attributed to these conditions. In essence, the diverse array of symptoms and dependence on various screening tools combine to create a landscape of uncertainty in the study of these conditions, casting potential shadows on the precision of prevalence estimates.

### THEORETICAL IMPLICATIONS FOR FUTURE RESEARCH

While acknowledging the possible limitations of the current study’s design, it is important to consider the theoretical implications of its findings and how they can be applied in designing future research on this subject. Although the current review is not necessarily representative of the entire global south, the resulting prevalence rates, as documented in the regions of interest, can probably be generalised for other populations in the global south.

First, the very fact that neuropsychiatric sequelae such as depression, anxiety, PTSD and OCD have significant prevalence rates in the global south challenges the previous narrative on the populations in these regions. Due to sociocultural views and the resultant idioms of distress, psychiatric disorders in this region are sometimes thought to be expressed differently compared to data obtained using diagnostic tools derived from international titles, such as the DSM and ICD. If these distinctions actually exist, their symptoms are likely to be considered ‘atypical’ and diagnosed as an indistinct ‘not otherwise specified’ subtype of the disorder.^105^ While it is clear that the existing literature challenges this perspective, concerted efforts are needed to develop disease-specific and culturally adaptive tools to identify post-TBI psychiatric disorders. Furthermore, more studies that use standardised clinical interviews, instead of self-report measures, would result in better comparability and reliability of results.

Second, despite the large population residing in West Asia, South Asia and Africa, the normative data for different populations in the global south have yet to be charted.^106^ Future studies in the global south should attempt to employ conventional and validated neuropsychological batteries to diagnose cognitive impairment. However, these high-power cognitive tests do not appear to be widely accessible to clinicians and researchers in these regions, as most of them are either not available in the public domain or, if available, require exorbitant fees that are not feasible for clinicians in certain resource-depleted regions.^107^ Thus, the allocation of resources for complications related to TBI is yet to receive due attention. Neuropsychological tests that are frequently used in the global south in the context of TBI are often not supported by relevant literature on their cross-cultural validity.^108^ Efforts are needed to unravel the relationship between cognitive symptoms and the critical neural substrates involved in cognition. This has the potential to lay the groundwork for the establishment of demographically valid and diseasespecific measures for cognition without engaging in a race-norming discourse on cognitive testing.^108^

Third, the interest in developing evidence-based rehabilitation and remediation for post-TBI conditions is increasing globally. There is evidence to suggest the efficacy of some of the pharmaco- and psycho-therapeutic interventions for post-TBI neuropsychiatric sequelae that were examined in the current review.^109^–^112^ Proper attention must be paid to adapting rehabilitation services for the TBI population in the global south.

Fourth, some of the articles that met the inclusion criteria were not featured in dominant search engines such as PsycINFO, Scopus, PubMed/MEDLINE and ProQuest. It is not clear whether more inclusive criteria would entail the potential consideration of articles published in journals that are sometimes labelled as ‘predatory’. Despite this caveat, such articles appeared to perform well with the inclusion criteria and screening using the JBI guidelines, with scores of greater than 75%, which, although adequate, falls within the lower range of quality control scores. However, such a threshold seems to be the best way to accumulate enough articles from the region of interest for a proper meta-analysis. In this regard, it appears that the North-South divide in terms of the quality and quantity of articles is evident in the research on the neuropsychiatric sequelae of TBI.^31^ The authors hope that the current critical appraisal of the literature from Western Asia, South Asia and Africa would be catalytic in addressing the unmet needs of those with brain injuries.

Fifth, this study employed the JBI guidelines to evaluate the quality of studies, which helped the authors select studies that adhered to more standardised methodologies. However, in future research, it is recommended that more standardised assessment tools and methodologies be used to improve the comparability and reliability of the findings of different studies. In the global south, access to healthcare resources is limited, with varying levels of awareness of neuropsychiatric sequelae and differences in reporting practises. These factors could have contributed to the observed prevalence rates. Therefore, to better understand these influences, more research involving qualitative investigations and sub-analyses could be conducted to explore the relationship between healthcare disparities and prevalence rates.

Finally, it should be noted that this study reported substantial prevalence rates for depression, anxiety, PTSD, OCD, TBI-SD and cognitive impairment following TBI in the specified regions. While existing studies lack homogeneous data, the consistency of these prevalence rates suggests a notable burden of neuropsychiatric sequelae of TBI in the ‘global south’. These findings underscore the need for targeted interventions, remedial services, neurorehabilitation and increasing awareness in the global south. Future research may investigate potential socioeconomic, cultural and contextual factors that could contribute to the observed patterns in this region, helping in the development of more tailored strategies for TBI prevention and management.

## Conclusion

To the best of the authors’ knowledge, this is the first critical review to examine the spectrum of post-TBI neuropsychiatric sequelae in the global south. The observed prevalence rates are significant and comparable to statistics from the global north. This challenges the existing narrative on the existence and presentation of neuropsychiatric symptoms among the populations of the global south and can help lay the foundation for the adaptation of rehabilitation services for patients with TBI in this region. Future studies should prioritise uniform assessment tools and methodologies for enhanced comparability. The limited access to healthcare, variations in awareness and reporting disparities in the global south could influence the prevalence rates of post-TBI neuropsychiatric sequelae, warranting qualitative investigations. The consistent prevalence rates of post-TBI neuropsychiatric sequelae in the included studies despite the heterogeneity of the data highlight their significant burden. This emphasises the need for targeted interventions, neurorehabilitation and increased awareness in the global south. Future efforts should explore socioeconomic, cultural and contextual factors to aid the development of tailored TBI prevention and management strategies.

## Supplementary Information



## Data Availability

This is a research article and all the data generated and analysed during this study have been included in this published article. Any raw data acquired can be provided upon reasonable request.
